# Myeloid derived suppressor cells – a new therapeutic target in the treatment of cancer

**DOI:** 10.1186/2051-1426-1-10

**Published:** 2013-07-15

**Authors:** Robert Wesolowski, Joseph Markowitz, William E Carson

**Affiliations:** 1 Division of Medical Oncology, B401 Starling Loving Hall, W10th Avenue, Columbus, OH 43210, USA; 2 Division of Medical Oncology, 406C Starling Loving Hall 320 W 10th Ave, Columbus, OH 43210, USA; 3 The Ohio State University Comprehensie Cancer Center, N911 Doan Hall, 410 West 10th Avenue, Columbus, OH 43210, USA

**Keywords:** Myeloid derived suppressor cells, Immunotherapy, Tumor immunology, Cancer vaccines

## Abstract

Myeloid Derived Suppressor Cells (MDSC) are a heterogeneous population of immature myeloid cells that are increased in states of cancer, inflammation and infection. In malignant states, MDSC are induced by tumor secreted growth factors. MDSC play an important part in suppression of host immune responses through several mechanisms such as production of arginase 1, release of reactive oxygen species and nitric oxide and secretion of immune-suppressive cytokines. This leads to a permissive immune environment necessary for the growth of malignant cells. MDSC may also contribute to angiogenesis and tumor invasion. This review focuses on currently available strategies to inhibit MDSC in the treatment of cancer.

## Introduction

Myeloid derived suppressor cells (MDSC) are a population of early myeloid cells that are expanded in various disease states including cancer and are capable of suppressing the immune response [1,2]. In mice, MDSC express myeloid markers (Gr1 or CD11b). In humans, the Gr1 antigen is absent. Human MDSC express myeloid cell markers such as CD11b^+^and CD33^+^, but are usually negative for HLA-DR and lineage specific antigens (Lin) such as CD3, CD19 and CD57. Monocytic MDSC are usually characterized by HLA-DR-, CD11b+, CD33+ and CD14^+^ phenotype in humans (CD11b + Ly6G^-^/Ly6C^+^ in mice) whereas mature monocytes express HLA-DR. Granulocytic MDSC are usually characterized by HLA-DR-, CD11b+, CD33+, CD15+ phenotype in humans (CD11b + Ly6G^+^/Ly6C^low^ in mice). The immune cells with these phenotypes have been shown to possess immunosuppressive properties [3-5]. The prevalence of MDSC immunophenotypes vary depending on the disease being studied [2,3]. An extensive discussion of MDSC phenotypic and functional heterogeneity is outside of the scope of this article and recent excellent reviews on this topic exist [1-3].

**Figure 1:**
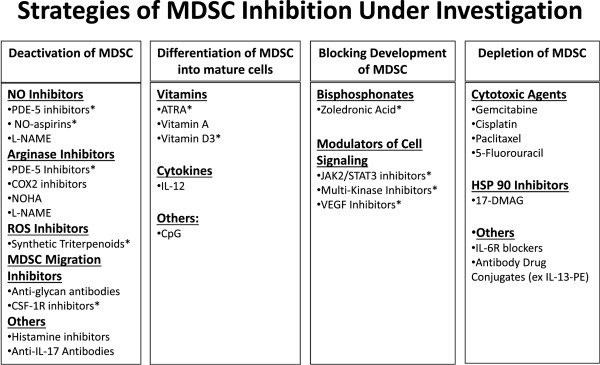
**Graphical representation of MDSC inhibition strategies. **(Abbreviations: NO – Nitric Oxide; PDE-5 – Phosphodiesterase 5; NO-Aspirin – Nitro-aspirin; L-Name – N(G)-Nitro-L-Arginine Methyl Ester; COX2 – Cyclooxygenase 2; CSF-1R – Colony Stimulating Factor Receptor 1; ATRA – All Trans Retinoic Acid; CpG – Deoxycytosine-Deoxyguanine Dinucleotide; JAK2 – Janus-Activated Kinase-2; STAT3 – Signal Transducer and Activator of Transcription 3; VEGF – Vascular Endothelial Growth Factor; 17-DMAG – 17-Dimethylaminoethylamino-17-Demethoxygeldanamycin; IL-6R – IL-6 Receptor); * – Agents that are presently under clinical investigation as MDSC inhibitors.

MDSC can be generated in the bone marrow in response to cancer derived factors such as granulocyte colony stimulating factor (G-CSF), IL-6, granulocyte monocyte colony stimulating factor (GM-CSF), IL-1β, prostaglandin E2 (PGE2), tumor necrosis factor α (TNFα) and vascular endothelial growth factor (VEGF) and are recruited to the tumor site by CCL2, CXCL12, and CXCL5 [6]. Additional signals stimulate MDSC to acquire immunosuppressive properties which are mediated through members of the signal transducer and activator of transcription (STAT1, STAT3, STAT6) and nuclear factor kappa-light-chain-enhancer of activated B cells (NF-κB) transcription factors [1]. Activated MDSC produce arginase 1 (ARG1), inducible nitric oxide synthase (NOS2), IDO (indoleamine 2,3-dioxygenase), NADPH oxidase and immunosuppressive cytokines that have the potential to inhibit cytotoxic T lymphocytes (CTLs), dendritic cells (DC), and natural killer (NK) cells as well as expand CD4^+^CD25^+^FoxP3^+^ regulatory T cells (Tregs). This leads to an immunologically permissive tumor microenvironment [7,8]. Peripheral blood MDSC levels correlate with a higher tumor burden and a worse prognosis [9-11]. MDSC may impair the efficacy of cancer vaccines via direct effects on T cell activation and antigen presentation by DC [12]. Inhibition of MDSC in murine models may enhance anti-tumor immunity by increasing responsiveness to interferon stimulation [13]. Inhibition or depletion of MDSC enhances the activity of cancer vaccines in animal models (Table 1). MDSC inhibition could be a useful adjunct to immune therapies in man and can be placed into four categories; 1) Deactivation of MDSC; 2) Differentiation of MDSC into mature cells; 3) Inhibition of myeloid cell development into MDSC; and 4) Depletion of MDSC (Figure 1). This review was undertaken to help researchers and clinicians become familiar with the many agents that can modulate MDSC function. Asterisk (*) was placed in Figure 1 next to the agents that are already undergoing clinical investigation as potential MDSC inhibitors in humans.

**Table 1:** Murine cancer vaccine studies that utilized MDSC inhibitors

**MDSC Inhibition Strategy**	**Tumor model**	**Vaccine**	**Effect of MDSC Modulation**
ATRA [14]	(a) C3 fibrosarcoma in C57BL/6 mice	(a) H-2D^b^-restricted epitope of the HPV-16 E7	(a) Decreased tumor size by ~3 fold at 35 days; enhanced CD8^+^ response
	(b) 3-methylcholanthrene-induced sarcoma containing mutant p53 gene in BALB/c mice	(b) Wild type p53 DC vaccine	(b) Decreased tumor size by ~5 fold; enhanced CD8+ responses
Gemcitabine [15]	Pancreas adenocarcinoma (Panc02) expressing murine survivin in C57BL/6 mice	Modified Vaccinia Ankara virus (MVA) expressing murine survivin protein	50% survival vs. 0% in control mice at 50 days; enhanced CD8^+^ responses
ATRA + anti-CD25 antibody [16]	Tumor bearing IL-1RI competent or IL-1RI deficient mice	cDNA IL-1α attenuated *S. typhimurium* and/or IL-1 competent or IL-1 deficient fibrosarcoma cell lysates	Decreased MDSC and Treg levels; significantly enhanced survival of IL-1 RI competent mice
Nitroaspirin derivative (NCX-4016) [17]	(a) CT26 colon carcinoma in C57BL/6 and BALB/c mice	Plasmid DNA vaccine encoding extracellular and trans-membrane domains of p185 peptide	(a) 20% cure rate at 120 days
	(b) Her-2/*neu* + (p185) N2C breast carcinoma in C57BL/6 and BALB/c mice		(b) 56% cure rate at 120 days
CDDO-Me (Triterpenoid) [18]	EL-4 thymoma in C57BL/6	DC transduced with murine survivin	Decreased tumor size by 2 fold; enhanced antigen specific immune response
IL-13-PE (cytotoxin composed of IL-13 and *Pseudomonas* exotoxin) [19]	(a) 4 T1 breast carcinoma in BALB/c mice	DNA vaccine encoding α2 chain of IL-13R	Decreased tumor size by 5 fold; decreased MDSC and Treg levels; enhanced T cell responses; enhanced survival by 35 days.
	(b) MCA304 sarcoma in C57BL/6 mice		
Sunitinib [20]	MO5 (B16.OVA: H-2^b^) melanoma on C57BL/6 mice	IL-12 transfected DC pulsed with OVA I and II peptides.	Loss of tumor associated MDSC and Tregs; enhanced CD8^+^ T cell responses
Gemcitabine [21]	Her-2/*neu* + SK-BR-3 breast carcinoma or mHER2/CT26 (colon carcinoma transfected with murine Her-2/*neu*) in BALB/c mice	(a) AdhHM	(a) No anti-tumor effect of AdhHM alone
		(b) AdhHM + anti- GITR antibody	(b) Decreased tumor size by >5 fold; (p < 0.005); CD8^+^ cell dependent rejection of syngeneic tumor cells
		(c) AdhHM + α galactosylceramide loaded DCs	(c) Decreased tumor size by >5 fold
Cisplatin [22]	TC-1 lung carcinoma expressing E7 protein in C57BL/6 mice	E7 DNA vaccine	Enhanced tumor lysis mediated by E7 specific CD8^+ ^cells; reduced tumor volume
Zoledronic Acid [23]	Transgenic Balb T-Neu mice (express activated rat *c-erbB-2/neu *transgene)	Plasmid DNA encoding portion of the rat *p185/Her-2 *gene	Delayed tumor onset and reduced in tumor size

Abbreviations: ATRA – All-Trans Retinoic Acid, AdhHM - Adenoviral vector expressing xenogenic human Her-2/neu, DC – Dendritic Cell, GITR - Glucocorticoid Induced Tumor Necrosis Factor Receptor Family- Related Receptor.

## Review

### Strategies for MDSC deactivation

#### ***Phosphodiesterase-5 inhibitors deactivate MDSC by interfering with arginase 1 and nitric oxide synthase expression***

Phosphodiesterase-5 (PDE-5) inhibitors such as sildenafil and tadalafil inhibit the degradation of cyclic guanosine monophosphate (cGMP) leading to reduction in ARG1 and NOS2 expression [24]. It is not entirely clear how this occurs but it has been proposed that high cGMP levels could interfere with expression of IL-4Rα on CD11b^+^ myeloid cells leading to decreased STAT6 activity and reduced levels of NOS2 and ARG1 [24]. ARG1 mediated depletion of L-arginine from the tumor microenvironment may be one of the mechanisms of MDSC induced T cell suppression secondary to decreased expression of the ζ subunit of CD3 [25]. MDSC also decrease the concentration of cysteine and tryptophan required for T cell activity [26]. However, some groups argue that IL4Rα is not involved in this process [27]. Proposed alternative mechanisms by which PDE-5 inhibitors function include destabilizing NOS2 mRNA, and decreasing cytosolic calcium concentration thereby reducing calcium-dependent protein kinase C signal transduction [24]. Treatment of mice with a PDE-5 inhibitor reduces the ability of MDSC to inhibit CD8^+^ T cells and leads to delayed tumor growth in mice inoculated with CT26WT colon carcinoma cells (a sign that T cell-mediated immunity might have been restored). The expansion of T cells within the peripheral blood mononuclear cell (PBMC) fraction isolated from patients with head and neck cancer and multiple myeloma and stimulated with anti-CD3/CD28 antibody–coated beads was enhanced following *in-vitro* treatment of PBMC with sildenafil [24].

Several clinical studies with PDE-5 inhibitors have been initiated. A phase II study in multiple myeloma is testing whether tadalafil can improve the response to lenalidomide and dexamethasone (NCT01374217 on http://www.clinicaltrials.gov). Other studies are testing whether neo-adjuvant tadalafil treatment in patients with oropharyngeal carcinoma can improve the infiltration of CD4^+^ and CD8^+^ cells into tumors (NCT00843635). A randomized trial of systemic chemotherapy with or without sildenafil in patients with non-small cell lung carcinoma (NCT00752115) has completed enrollment, while a phase I trial in pancreatic cancer is testing tadalafil and a telomerase vaccine alongside with gemcitabine chemotherapy followed by low dose gemcitabine and radiation therapy (NCT01342224).

#### ***Nitro-aspirin (NO-aspirin) interferes with MDSC nitric oxide metabolism***

Decreased T cell responsiveness in the presence of MDSC may be a direct result of nitric oxide production by MDSC leading to increased nitration of the T cell receptor, CCL2 or STAT1 [13,28,29]. NO-aspirins suppress the production of reactive oxygen species (ROS) and provide feedback inhibition of NOS2 [30]. A potent NO-aspirin, AT-38 (([3-(aminocarbonyl)furoxan-4-yl]methyl salicylate) inhibits inducible NOS via various mechanisms and leads to the reversal of MDSC induced inhibition of T-cell responses *in-vitro* by reducing CCL2 chemokine nitration [29]. Treatment of C26 colon carcinoma-bearing mice with a nitro-aspirin, NCX-4016 (2-(acetyloxy)benzoic acid 3-(nitrooxymethyl)phenyl ester) improved T cell proliferation, decreased numbers of MDSC within the tumor, and retarded tumor growth compared to control animals [17]. De Santo *et al.* challenged mice with a DNA vaccine encoding the endogenous retro-viral envelope glycoprotein gp70 (gp70env) and established CT26 tumors that express gp70env. The mice were treated with NO-aspirin on days 1–18 following tumor challenge, which resulted in significantly longer survival of the vaccinated animals. At 120 days, 20% of animals treated with NCX-4016 and the vaccine did not develop tumors, whereas all mice that received either NCX-4016 or the vaccine alone developed tumors [17]. NCX-4016 therapy is presently under investigation in a phase I clinical trial for prevention of colorectal cancer in patients at high risk of developing this malignancy (NCT00331786).

#### ***Synthetic triterpenoids deactivate MDSC by reducing reactive oxygen species (ROS)***

Bardoxolone methyl (CDDO-Me), a synthetic triterpenoid, is a methyl ester of 2-cyano-3,12-dioxooleana-1,9(11)-dien-28-oic acid and is a potent activator of the nuclear factor-erythroid 2-related factor 2 (NFR2) transcription factor. NFR2 leads to up-regulation of antioxidant genes, including NADPH: quinone oxidoreductase 1 (NQO1), and thioredoxin [31]. At higher concentrations (1–5 μM), CDDO-Me can inhibit STAT3, reducing the expansion of MDSC [1,32]. CDDO-Me treatment of Gr1^+^/CD11b^+^ splenocytes isolated from mice bearing the EL-4 thymoma caused up-regulation of the anti-oxidant enzyme NQO1, decreased concentrations of ROS, and led to reduced levels of nitro-tyrosine residues inside MDSC. CDDO-Me treatment of tumor-bearing mice led to decreased MDSC production of ROS, improvement in T cell function and reduced tumor growth. In an allogeneic mixed leukocyte reaction, MDSC-mediated inhibition of T cells from patients with renal cell carcinoma was abrogated by CDDO-Me at a concentration of 200–300 nM, which is achievable *in vivo*. CDDO-Me treatment of mice bearing EL-4 tumors greatly enhanced the anti-tumor effects of a vaccine consisting of survivin-transduced DC and this was associated with a robust T cell response to re-stimulation with a survivin-derived peptide [18]. CDDO-Me treatment of pancreatic cancer patients receiving gemcitabine in a phase I clinical trial led to significantly increased T cell responses to tetanus toxoid and phytohemagglutinin (Clinical Trial No. RTA 402-C-0702).

#### ***Cyclooxygenase 2 (COX2) inhibitors reduce MDSC suppressive function by decreasing expression of Arginase 1***

MDSC have increased expression of the Prostaglandin E (PGE) receptor. Treatment of tumor-bearing mice with a COX-2 inhibitor led to an approximate 50% reduction in tumor growth rate and decreased levels of Gr1^+^/CD11b^+^ cells. These results were confirmed in PGE2 receptor knockout mice [33]. Rodriguez *et al.* showed that PGE2 produced by 3LL lung carcinoma cells was able to induce ARG1 expression in tumor-associated MDSC [34]. Treatment of 3LL tumor-bearing mice with the COX-2 inhibitor sc-58125 led to complete blockade of ARG1 expression in the tumor and a statistically significant decrease in tumor volume (compared to untreated tumor baring animals), an effect that was not observed in immune-deficient mice. A widely used COX-2 inhibitor, celecoxib, was administered to mice that had been treated with 1,2-dimethylhydrazine diHCl to stimulate the development of colon cancer. Celecoxib use was associated with lower levels of Gr1^+^/CD11b^+^ myeloid cells and higher numbers of tumor infiltrating lymphocytes [35]. COX2 inhibitors may therefore have more than one mechanism of suppressing MDSC, namely they can block their activation and also reduce their numbers [36].

#### ***Other arginase inhibitors***

One of the most potent physiologic inhibitors of ARG1 activity is N-hydroxy-L-Arginine (NOHA), an oxidized form of arginine that is an intermediate in the enzymatic conversion of arginine to citruline and nitric oxide by NOS2 [37]. This compound has been used as an ARG inhibitor in animal and *in vitro* studies where inhibition of MDSC function was desired. For example, in experiments involving the A20 B-cell lymphoma line, use of NOHA effectively inhibited MDSC mediated expansion of Tregs and eliminated tumor induced immune tolerance [38].

N(G)-Nitro-L-Arginine Methyl Ester (L-NAME) is another compound that has been shown to inhibit Arg 1 activity [39]. In addition, L-NAME is a transcriptional downregulator of NOS which leads to reduced production of nitric oxide [40]. L-NAME was shown to decrease immunosuppressive MDSC activity in C57BL/6 mice bearing the C26GM colon carcinoma and RMA T lymphoma cells leading to slower tumor growth and improved tumor specific immune responses in the treated animals [41]. Regamonti and colleagues were able to demonstrate that treatment of C57BL/6 mice implanted with TRAMP-C1 prostate cancer cells with L-NAME resulted in reduction in the immunosuppressive action of CD11b^+^ myeloid cells (including inhibition of Arg1 activity) in the spleen and within the tumor. The treatment also improved survival of the treated animals (50% of tumor bearing mice were alive at the time when all vehicle treated mice had died from tumor overgrowth at about 36 days post implantation). However, the agent did not inhibit tumor progression or break the tumor specific tolerance of a transgenic murine model that spontaneously developed prostate adenocarcinoma. *In-vitro* assays showed an inability of activated CD8+ T-cells derived from the spleens of L-NAME treated animals to lyse syngeneic target tumor cells [42].

#### ***Anti-glycan antibody inhibits the migration of MDSC***

The Receptor for Advanced Glycation End Products (RAGE) is modified post-translationally by the addition of carboxylated glycans at several amino-acid residues. RAGE is present on the membrane of colon carcinomas, while one of its ligands, S100A8/A9 is expressed on Gr1^+^/CD11b^+^ murine MDSC. S100A8/A9 is induced via STAT3 mediated signaling and appears to be involved in upregulation of MDSC in cancer and the differentiation of dendritic cells and macrophages [43]. Interference with S100A8/A9 signaling can result in inhibition of MDSC function leading to decreased tumor growth. This has been demonstrated in experiments involving mice that develop colorectal cancer in response to treatment with azoxymethane (AOM) and exposure to the inflammatory agent dextran sodium sulfate (DSS) (AOM-DSS mice). AOM-DSS mice treated with the anti-glycan mAbGB3.1 demonstrated a 75% reduction in the formation of colonic tumors and decreased serum levels of NF-κB-induced cytokines such as TNFα and IL-6. This result suggested that RAGE and S100A8/A9 form a feedback loop that could play a role in promoting MDSC recruitment to colon cancers and provides a rationale for the clinical use of anti-glycan antibodies [44]. Sinha *et al.* have shown that RAGE and other cell surface glycoproteins may be present on MDSC. MDSC expression of S100A8/A9 can therefore complete an autocrine loop that leads to enhanced MDSC accumulation. This RAGE-S100 signaling loop may activate the NF-κB transcription factor, which suggests that NF-κB inhibitors might also be an effective means of blocking MDSC activity [45].

#### ***Inhibitors of colony stimulating factors and their receptors block the migration of MDSC***

Colony stimulating factor receptor 1 (CSF-1R) may play a role in recruitment of MDSC to tumor sites and the induction of angiogenesis. BALB/c mice bearing C26GM colon carcinoma tumors that secrete GM-CSF exhibit high levels of MDSC in the spleen and tumor [17]. Treatment of mice bearing Lewis Lung Carcinoma tumors with a small molecule inhibitor of CSF-1R (GW2580: 5-(3-Methoxy-4-((4-methoxybenzyl)oxy)benzyl)pyrimidine-2,4-diamine) inhibited the recruitment of CD11b^+^Gr1^lo^Ly6C^hi^ monocytic MDSC into tumors and reduced the expression of pro-angiogenic and immunosuppressive genes within the tumor microenvironment [46]. Xu and colleagues demonstrated that tumor irradiation of C57BL/6 mice implanted with syngeneic RM-1 prostate cancer cells led to an increase in CSF1 expression by the tumor cells, which was dependent on activation of ABL-1 tyrosine kinase. This further led to infiltration of the tumors by the tumor associated macrophages (TAM) as well as monocytic and granulocytic MDSC. Blocking CSF-1R with GW2580 or PLX3397 following tumor irradiation resulted in a decrease of myeloid cell infiltration (both TAM and MDSC) in the tumors and spleens and led to a slower tumor growth compared to irradiated animas not treated with CSF-1R inhibitor [47]. Monoclonal antibodies that block the CSF-1R (e.g. IMC-CS4) as well as small molecule inhibitors of CSF-1R (e.g. PLX-3397) are undergoing phase I clinical trials.

#### ***Histamine and MDSC***

It has been shown that histamine may stimulate GM-CSF and IL-6 production via histamine H_1_ and histamine H_2_ receptors on human PBMC *in-vitro*[48]. However, more recently, in an *in-vivo* model by Yang *et al*., it has been shown that a deficiency of histamine directly stimulates the production of CD11b^+^Ly6G^+^ early myeloid cells in murine models of skin and colon carcinogenesis [49]. In the commentary to this article, it was described that histamine is approved as an agent for AML in Europe and Israel and has been found to have anti-tumor effects in melanoma, lymphoma, fibrosarcoma and colon cancer [50]. H2 blockers such as cimetidine appear to induce apoptosis of MDSC through induction of Fas and FasL [51].

#### ***Other potential approaches to inhibit MDSC activation***

IL-17 also seems to be important for recruiting of MDSC to tumor sites in murine models [52]. In experiments involving tumor-bearing mice that were deficient in IL-17R and IFNγR, tumor development was inhibited and this was associated with increased cytotoxic T cell infiltration of tumors and lower MDSC levels. Also, treatment of tumor-bearing wild type mice with neutralizing anti-IL-17 antibody led to decreased tumor growth. Conversely, IL-17 treatment promoted tumor growth and MDSC infiltration of tumors [52].

### MDSC differentiating agents

#### ***All-trans retinoic acid***

All-trans retinoic acid (ATRA) is a metabolite of vitamin A with agonistic activity towards nuclear receptors that are retinoid–activated transcriptional regulators (RARα, RARβ, etc.) [53]. These factors activate target genes that lead to maturation of early myeloid cells into their fully differentiated (and hence less-immunosuppressive) forms [54]. MDSC isolated from the peripheral blood of patients with advanced renal cell carcinoma express RARα and RARγ nuclear receptors. Treatment of MDSC with ATRA led to increased MDSC expression of differentiation markers such as HLA-DR [55]. Adoptive transfer experiments in tumor-bearing animals showed that ATRA can lead to differentiation of MDSC into DC, granulocytes and monocytes with concomitant improvement of CTL-mediated immune responses [14]. ATRA administration improves vaccine therapy in several murine models. Mice treated with a vaccine targeting the H-2D^b^-restricted epitope of the Human Papilloma Virus 16 (HPV-16) E7 protein after C3 fibrosarcoma tumor implantation and exposed to ATRA displayed a 3 fold decrease in tumor growth and improved splenocyte IFNγ production. Similarly, BALB/c mice inoculated with immunogenic 3-methylcholantrene-induced sarcomas expressing a mutant p53 gene, and treated with wild type p53 primed DC had 5 fold smaller tumors after ATRA treatment. Only T cells isolated from the spleens of ATRA treated, immunized mice demonstrated a significant antigen-specific immune response 4 weeks after tumor inoculation [14].

In one study, treatment of 18 renal cell carcinoma patients with ATRA for 7 days led to a significant reduction in peripheral blood MDSC. Patients who achieved high ATRA plasma concentrations had a decline in peripheral blood MDSC to levels seen in healthy control subjects. This was associated with an improvement in the plasmacytoid to myeloid DC ratio, higher IFNγ and IL-2 plasma levels, and an increased type 1 to type 2 T-helper cell ratio (Th1/Th2) [56]. Two clinical trials that employ ATRA to modulate MDSC are currently in process. A phase II trial in patients with lung adenocarcinoma resistant to chemotherapy is testing an allogeneic tumor based-cell vaccine and the cytotoxic agent cyclophosphamide in combination with ATRA (NCT00601796). And a randomized phase II trial is testing whether ATRA can enhance the efficacy of chemotherapy combined with a vaccine consisting of DC transduced with a p53 expressing adenoviral vector in patients with extensive stage small cell lung cancer (NCT00618891).

#### ***Vitamins***

Vitamins such as Vitamin D3 or Vitamin A may also enhance maturation of myeloid cells. *In-vitro* studies show that these vitamins decrease levels of immature myeloid cells by inducing their maturation and lead to improved anti-tumor activity in the context of immunotherapeutic interventions [1,57]. A study with 25-hydroxy-vitamin D was conducted in patients with squamous cell carcinoma of head and neck. Patients receiving the highest dose (60 μg/day) had significantly increased expression of HLA-DR on myeloid cells and increased blood levels of IL-12 and IFNγ [58].

#### ***Other differentiating agents***

DNA fragments that contain a high frequency of unmethylated deoxycytosine-deoxyguanine dinucleotide (CpG) motifs (common in bacterial and viral DNA) can stimulate immune cells via Toll-like receptor 9 (TLR9). TLR9 is expressed on DC, B cells, monocytes and NK cells. Stimulation of TLR9 activates the immune response through increased production of IL-12 and type I interferons [59]. Treatment of mice with TLR9 ligand agonists such as CpG oligodeoxynucleotides (ODN) decreased the prevalence of the LY6G^hi^ MDSC subset. CpG ODN promoted the increased production of IFNα by cytoplasmoid DC, which is thought to mediate maturation of MDSC. Administration of IFNα alone can abrogate MDSC mediated T cell inhibition [60].

Macrophage-mediated IL-12 production can be inhibited by MDSCs in chronic inflammation via activation of a TLR4 signaling pathway [61]. In experiments with a spontaneously metastasizing 4T1 mouse mammary carcinoma cells, MDSC were found to suppress Th1 immunity. MDSC interacted with macrophages leading to decreased production of IL-12 and increased production of IL-10. The result was the predominance of a Th2 immune response favorable to cancer growth [61]. In a murine model of breast cancer, mice treated with IL-12 exhibited differentiation of MDSC at tumor sites, increased overall survival, decreased lung metastasis, and reduced levels of mRNA encoding NO2 and IFNγ [62]. In another study, lymphodepleted mice bearing subcutaneous tumors were treated with syngeneic T cells that were co-transduced with an anti-VEGFR-2 (Vascular Endothelial Growth Factor Receptor 2) chimeric antigen receptor (CAR) and constitutively expressed single-chain murine IL-12. Administration of these T cells to the tumor bearing mice led to retardation of tumor growth and inhibition of systemic and intratumoral CD11b^+^Gr1^+^VEGFR-2^+^ myeloid suppressor cells [63]. However, it was shown that the anti-tumor effects of IL-12 were not dependent upon direct binding of IL-12 to receptors on lymphocytes or NK cells. Instead, IL-12 indirectly enhanced the activity of adoptively transferred CD8^+^ T cells by affecting bone marrow–derived tumor macrophages, dendritic cells, and myeloid–derived suppressor cells [64].

### Agents that block the formation of MDSC

#### ***Nitro-Bisphosphonates (N-Bisphosphonates)***

N-Bisphosphonates inhibit bone resorbing osteoclasts [65]. They inhibit the enzyme farnesyl-diphosphate (FPP) synthase which is responsible for the generation of geranyl and prenyl compounds that are added to many proteins as post-translational modifications [65]. Treatment with N-Bisphosphonate leads to decreased prenylation of proteins such as matrix metalloproteinase 9 (MMP9). MMP9 may influence MDSC generation/function by cleaving c-kit, which is believed to play a role in MDSC mobilization from the bone marrow niche [66]. MMP9 has also been found to mobilize VEGF thereby making it available to bind to its receptor (VEGFR) on MDSC [23]. N-bisphosphonate treatment of transgenic BALB-neuT mice (expressing an activated rat *c-erbB-2/neu* transgene) that develop metastatic mammary carcinomas resulted in lower levels of MDSC and lower tumor burden compared to untreated control animals. Likewise, use of zoledronic acid with a plasmid DNA vaccine encoding rat p185/Her-2 resulted in delayed tumor growth and the increased induction of anti-p185/Her-2 antibodies as compared to controls [23]. Porembka *et al.* studied the effects of the bisphosphonate zoledronic acid on mice inoculated with the pancreatic cancer cell line Panc02. Animals treated with zoledronic acid demonstrated less intra-tumoral MDSC accumulation, and this was associated with delayed tumor growth rate, prolonged median survival, and increased recruitment of T cells to the tumor. Zoledronic acid treated mice also had increased levels of IFNγ and decreased levels of IL-10 within the tumors [67]. Wesolowski and colleagues are presently studying the effects of zoledronic acid on MDSC levels in patients with estrogen receptor positive breast cancer who are receiving endocrine therapy.

#### ***Modulators of cell signaling***

Tyrosine kinase signaling has been implicated in the stimulation of early myeloid cell differentiation into MDSC. Constitutive activation of STAT3 in MDSC upregulates anti-apoptotic, pro-proliferative, and pro-angiogenic genes [1,68]. Inhibitors of STAT3 activation such as peptidomimetics, small molecule inhibitors and platinum agents have been employed against MDSC [69]. Derivatives of curcurmin, have been synthesized and have been shown to inhibit STAT3 phosphorylation and subsequent activation [70,71]. Administration of Cucurbitacin B (CuB), a selective inhibitor of the JAK2/STAT3 pathway, to patients with advanced lung cancers decreased peripheral blood levels of Lin^-^HLA-DR^-^CD33^+^ immature myeloid cells and increased peripheral blood levels of Lin^-^HLA-DR^+^CD33^+^ mature myeloid cells compared with baseline levels. *In vitro* experiments demonstrated that CuB induced differentiation of DC and increased the sensitivity of the tumor cells to p53-specific CTL cells [72].

Sunitinib is a multi-kinase inhibitor that has multiple targets including VEGFR and c-kit. Treatment with sunitinib led to a 50% reduction in the peripheral blood levels of MDSC in renal cell cancer patients. The decline was associated with improved Th1 lymphocyte function and decreased numbers of Tregs [9]. In contrast, a phase I clinical trial of a VEGF trap demonstrated no effect on peripheral blood levels of MDSC [73]. Similarly, anti-VEGF antibody use in patients with renal cell carcinoma did not appear to reduce the levels of peripheral blood MDSC but did increase the levels of mature DCs [74]. This is a surprising finding, given that high levels of VEGF have been associated with the accumulation of immature myeloid DC in cancer patients [1]. However, the discrepancy between pre-clinical and early clinical research can be explained by the heterogeneous patient population participating in the latter, often with various end-stage and treatment refractory malignancies. In addition, these early phase clinical studies are often not powered to provide definitive conclusions based on correlative markers, as they are designed mainly to provide data on the safety of a study drug. This can make interpretation of such results difficult. Additional clinical research utilizing more homogeneous patient populations is warranted.

### Agents that decrease MDSC levels

Some cytotoxic agents have been found to cause MDSC depletion through as yet incompletely understood mechanisms. Gemcitabine treatment of tumor-bearing mice reduced the number of splenic and tumor Gr1^+^/CD11b^+^ MDSC without affecting the numbers of CD4^+^ or CD8^+^ T cells or NK cells [75]. Le *at al.* showed that weekly gemcitabine treatments reduced tumor size and levels of splenic MDSCs in mice bearing 4T1 tumors [76]. Gemcitabine treatment of mice bearing established Panc02 pancreatic adenocarcinomas prior to administration of a modified Vaccinia Ankara (MVA) based viral vaccine against the murine survivin protein led to the greatest reduction in tumor volume compared to controls [15]. Inhibition of MDSC accumulation with gemcitabine also enhanced the activity of a Her-2/*neu* adenoviral vector vaccine [21]. Other cytotoxic agents may also inhibit MDSC. Cisplatin treatment of C57BL/6 mice bearing TC-1 lung carcinomas resulted in reduced numbers of MDSC and Tregs compared to untreated animals [22]. 5-Fluorouracil (5-FU) treatment of EL-7 thymoma-bearing C57BL/6 mice has been found to lead to a reduction in splenic and tumor MDSC. This agent had no significant effect on other immune cells (T, DC, NK, NKT cells) except for an increase in the number of B cells [77]. Paclitaxel, an agent that inhibits disassembly of microtubules, may down-regulate the function of MDSC by causing them to differentiate into mature DC [78].

Other strategies for depletion of MDSCs are emerging. One approach involves the use of the heat shock protein 90 (HSP90) inhibitor, 17-DMAG (17-Dimethylaminoethylamino-17-demethoxygeldanamycin). When cells are treated with 17-DMAG, EphA2 (HSP90 client protein) is degraded by the proteasome. Tumor cells can then be recognized by Type-1 anti-EphA2 CD8+ cells. When an antibody to EphA2 and an inhibitor to HSP90 are combined in a sarcoma murine model, mice are rendered free of sarcoma and this has been associated with reduced numbers of MDSC [79]. Combination therapy with 17-DMAG apparently reconditions the tumor microenvironment improving the recruitment of anti-tumor T cells. It has been hypothesized that FAS ligand (FASL) positive T cells might be important in the regulation of MDSC survival. FAS(+) MDSCs were shown to be susceptible to FAS-mediated killing *in vitro*[80]. In addition, a recent study showed that blocking IL-6R, a key receptor to a cytokine that is associated with suppression of the cytotoxic immune response, may also lead to decreases in accumulation of monocytic and granulocytic MDSC, reduction in tumor growth and improvement in T-cell function in squamous cell carcinoma of the skin (CMC-1 cells) bearing tumor bearing mice (all compared to control tumor bearing animals). Gemcitabine used alongside with a monoclonal antibody against IL-6R led to greater downmodulation of MDSC compared to use of the monoclonal antibody alone [81].

Nakashima *et al.* depleted MDSC via the use of IL-13 linked to *Pseudomonas* exotoxin (IL-13-PE) in combination with a DNA vaccine against IL-13Rα2. They tested this combination therapy on C57BL/6 and BALB/c mice bearing established MCA307 sarcoma tumors and 4T1 breast carcinoma tumors that both naturally express the IL-13Rα. Mice that were treated with IL-13-PE followed by injection of the IL-13Rα2 DNA vaccine had a 5-fold greater decrease in tumor growth compared to the animals that received the vaccine alone. Treatment of mice with IL-13-PE and the IL-13Rα2 vaccine resulted in depletion of MDSC, increased numbers of CD8^+^ T cells and the release of IFNγ [19]. IL-13 may be playing crucial role in the MDSC-mediated T cell interaction and increased levels of IL-13 are associated with increased levels of MDSC [10]. Other strategies to deplete MDSC in murine experiments involved the use of antibodies against the Gr1 antigen. Treatment of tumor bearing mice with anti-Gr1+ antibodies resulted in retardation of tumor growth [1,7]. However, Gr1 is a general granulocyte marker and may also deplete neutrophils making this MDSC depleting strategy controversial. It is also important to note that Gr1 antigen is not present on human MDSC. However, these experiments suggest that depletion of MDSC could be an effective adjunct to immunotherapies in the clinical setting.

## Conclusion

Pre-clinical evidence suggests that cancer vaccines are more effective in tumor bearing mice that have been depleted of MDSC [14,17,18,23]. The overall mechanism of cancer mediated expansion of MDSC and the resultant immune suppression is expected to be similar between humans and mice making the results of murine experiments useful in developing new anti-MDSC agents and testing them in clinical trials. There is wide range of potential therapeutic targets that are involved in MDSC production and their immunosuppressive function. MDSC may be inhibited via the use of phosphodiesterase inhibitors, nitroaspirins, synthetic triterpenoids, COX2 inhibitors, ARG1 inhibitors, anti-glycan antibodies, CSF-1R, IL-17 inhibitors and histamine based approaches. MDSC may be differentiated by using ATRA, vitamins A or D3 or IL-12. Agents that block the formation of MDSC include N-Bisphosphonates, modulators of tyrosine kinases, and STAT3 inhibitors. Agents that decrease levels of MDSC include gemcitabine, HSP90 inhibitors, and paclitaxel. Some compounds, such as ATRA, PDE5 inhibitors, nitro-aspirins (e.x. NCX 4016), or tyrosine kinase inhibitors are already in clinical trials testing their ability to inhibit MDSC and enhance anti-tumor immunity in humans. Others such as anti-histamines, anti-glycan inhibitors, CpG, IL-12, IL-13-PE or HSP 90 inhibitors are still undergoing testing as MDSC inhibitors in pre-clinical models. Compounds already FDA approved (e.g. ATRA, PDE5 inhibitors, COX-2 inhibitors or bisphosphonates) will likely be the first to enter late phase clinical trials to test their ability to suppress MDSC and improve the efficacy of immune modulating therapies (immune checkpoint inhibitors or cancer vaccines). Further research is needed to identify the most promising compounds for clinical development.

## Abbreviations

5-FU: 5-Fluorouracil; AOM: Azoxymethane; ADC: Antibody drug conjugate; ARG: Arginase; ATRA: All-trans retinoic acid; CAR: Chimeric antigen receptor; CCL: Chemokine Cc motif Ligand; CD: Cluster of differentiation; CDDO-Me: Bardoxolone methyl; CSF-1R: Colony stimulating factor receptor 1; cGMP: Cyclic guanosine monophosphate; CpG: Unmethylated deoxycytosine-deoxyguanine dinucleotide (CpG) motifs; CpG ODN: Oligodexynucleotides; CTL: Cytotoxic T lymphocytes; CXCL: CXC Chemokine Ligand; COX2: Cyclooxygenase 2; CuB: Cucurbitacin B; DC: Dendritic cells; DSS: Dextran sodium sulfate; DNA: Deoxyribonucleic acid; EphA2: Ephrin type-A receptor 2; FasL: Fas ligand; FoxP3: Forkhead box P3; FPP: Farnesyl-diphosphate; G-CSF: Granulocyte colony stimulating factor; GM-CSF: Granulocyte monocyte colony stimulating factor; gp70env: Envelope glycoprotein gp70; Gr1: Granulocyte differentiation antigen 1; H2 blockers: Histamine H2 receptor blockers; Her-2/neu: Human epiderrmal growth factor receptor 2; HLA: Human leukocyte antigen; HPV: Human papilloma virus; HSP90: Heat shock protein 90; IDO: Indoleamine 2,3-Dioxygenase; IFN: Interferon; IFNγR: Interferon gamma receptor; IL: Interleukin; IL-13-PE: IL-13 Linked to pseudomonas exotoxin; IL4Rα: Interleukin 4 Receptor α chain; JAK2: Janus-Activated Kinase-2; Lin: Lineage marker; L-NAME: N(G)-Nitro-L-Arginine Methyl Ester; MDSC: Myeloid derived suppressor cell(s); mRNA: messenger ribonucleic acid; MMP9: Metalloproteinase 9; MVA: Modified vaccinia ankara; NADPH: Nicotinamide adenine dinucleotide phosphate (reduced).; NF-kB: Nuclear Factor kappa-light-chain-enhancer of activated B cells; NK: Natural Killer cells; NKT: Natural killer T cells; NO-aspirin: Nitro-aspirin; NOHA: N-hydroxy-L-Arginine; NOS: Nitric oxide synthase; NQO1: Quinone oxidoreductase 1; NRF2: Nuclear factor-erythroid 2-Related Factor 2; PBMC: Peripheral blood mononuclear cells; PGE: Prostaglandin E; PGE-5: Phosphodiesterase-5; RAGE: Receptor for advanced Glycation End products; RAR: Retinoid-activated transcriptional regulators; ROS: Reactive oxygen species; STAT: Signal transducer and activator of transcription; Th1: Type 1 T-helper cells; Th2: Type 2 T-helper cells; TLR: Tall-Like Receptor; TNF: Tumor necrosis factor; Tregs: T regulatory cells; TRAMP: Transgenic adenocarcinoma of the mouse prostate; VEGF: Vascular endothelial growth factor; VEGFR-2: Vascular endothelial growth factor receptor 2.

## Competing interests

All authors declare that they have no competing interests.

## Authors’ contributions

RW performed the literature search; drafted and edited all versions of the manuscript; incorporated all edits from JM and WC. JM performed literature search, made substantial contributions to all drafts of the manuscripts both in terms of content and organization. Supervised preparation of the manuscript; contributed ideas and publications to be used in drafting the manuscript; read and edited all drafts of the manuscript. All authors read and approved the final manuscript.

## Authors’ information

RW is an Assistant Professor in Department of Internal Medicine at Ohio State University on a tenure track. Dr. Wesolowski is a board certified academic medical oncologist involved in translational and clinical research and treatment of breast cancer. He currently collaborates with Dr. William Carson’s laboratory. His research interests are in tumor immunology, early phase clinical trials and prognostic and predictive value of Myeloid Derived Suppressor Cells (MDSC).

JM is a physician scientist, is a U.S. Board Certified Internist who has just completed a clinical Medical Oncology training program. Currently, he is working in the laboratory of Dr. William Carson as a postdoctoral fellow. To date, he has had experience both in pre-clinical and clinical development of experimental therapeutics including a published patent. He has expertise in biochemistry, protein NMR spectroscopy, and fluorescence spectroscopy. He has joined Dr. Carson’s laboratory to gain experience in immunology and animal tumor models.

WC is a Professor of Surgery and Medical Microbiology and Immunology. Dr. Carson is also an associate director of clinical research at the Ohio State University Comprehensive Cancer Center and a co-leader of the institution’s Immunology Program. He is co-Investigator on a Project Program Grant in Innate Immunity. Dr. Carson chairs the Institutional Review Board at the Ohio State University, and is the Institutional principal investigator for National Comprehensive Cancer Network. He has a laboratory and focuses on work related to exploring interactions of immune system and malignancies. He also specifically investigates immune function of MDSC. His work includes testing MDSC inhibitors and studying pre-clinical models of MDSC inhibition that would facilitate the development of early clinical trials with MDSC modulating agents.

Robert Wesolowski and Joseph Markowitz are co-first authors.
